# Chitin degradation potential and whole-genome sequence of *Streptomyces diastaticus* strain CS1801

**DOI:** 10.1186/s13568-020-0963-6

**Published:** 2020-02-08

**Authors:** Tiantian Xu, Manting Qi, Haiying Liu, Dan Cao, Chenlei Xu, Limei Wang, Bin Qi

**Affiliations:** 1 Research Center of Fermentation Engineering, Changshu Institute of Technology, Changshu, 215500 China; 2 College of Pharmaceutical Science, Soochow University, Suzhou, 215123 China; 3 Department of Biological Science, Xi’an Jiaotong-Liverpool University, Suzhou, 215123 China; 4 School of Food Science and Technology, Jiangnan University, Wuxi, 214122 China

**Keywords:** Chitin, Chitinase, Biodegradation, Whole genome, Crustacean waste utilization

## Abstract

The aim of this study was to evaluate the chitin degradation potential and whole-genome sequence of *Streptomyces diastaticus* strain CS1801, which had been screened out in our previous work. The results of fermentation revealed that CS1801 can convert the chitin derived from crab shells, colloidal chitin and *N*-acetylglucosamine to chitooligosaccharide. Additional genome-wide analysis of CS1801 was also performed to explore the genomic basis for chitin degradation. The results showed that CS1801 possesses a chromosome with 5,611,479 bp (73% GC) and a plasmid with 1,388,284 bp (73% GC). The CS1801 genome consists of 7584 protein-coding genes, 90 tRNA and 21 rRNA operons. In addition, the results of genomic CAZyme analysis indicated that CS1801 comprises 103 glycoside hydrolase family genes, which could regulate the glycoside hydrolases that contribute to chitin degradation. The whole-genome information of CS1801 could highlight the mechanism underlying the chitin degradation activity of CS1801, strongly indicating that CS1801 is characterized by a substantial number of genes encoding chitinases and the complete metabolic pathway of chitin, conferring CS1801 with promising potential applicability in chitooligosaccharide production.

## Introduction

Chitooligosaccharide (COS) is a water-soluble polysaccharide obtained by treatment of chitin or chitosan with acid hydrolysis, enzymatic degradation or both (Einbu et al. 2007; Liu et al. 2009). Generally, COS is characterized by a degree of polymerization (DP) of less than 20 (Lee et al. 2002). COS performs many functions, such as antibacterial effects (Sun et al. 2018), antioxidant activity (Fang et al. 2015), and animal and plant growth promotion (Nandhini et al. 2017; Shenghe et al. 2017). COS has become a research topic of interest and has been widely used in medicine (Zhao et al. 2017; Zhou et al. 2018), agriculture (Swiatkiewicz et al. 2015; Lan et al. 2016) and food (Cao et al. 2018; Jiang et al. 2018).

Chitin is an important precursor for the production of COS and a polymer of *N*-acetylglucosamine (GlcNAc) linked by β-1,4-glycosidic bonds (Nguyen-Thi and Doucet 2016). Chitin is ubiquitous; its reserves are second only to those of cellulose, which is the second largest renewable resource on Earth, and approximately 10 billion tons of chitin is biosynthesized (Rinaudo 2006). The main raw material used in the industrial production of chitin is discarded shrimp and crab shells from aquatic processing plants. These shells contain more than 20% chitin (Hamdi et al. 2017). In China, due to the large number of lakes and the vast sea area, shrimp and crab resources are abundant. With the development of the Chinese seafood and farmed shrimp and crab industries, the amount of these wastes is increasing, causing serious environmental pollution and increasing the burden on businesses. However, these wastes are important resources.

At present, COS preparation is performed largely via a chemical method. Enzyme-assisted hydrolysis is a better method to obtain COS than chemical methods, with higher purity and lower pollution, but the search for proper enzymes remains ongoing (Liang et al. 2018).

Chitinase and chitosanase are promising enzymes for the production of COS, which has been reported in the literature (Kidibule et al. 2018; Zhang et al. 2018; Guo et al. 2019). The gene encoding chitosanase from *Streptomyces albolongus* was cloned, sequenced and expressed in *Escherichia coli* and shown to hydrolyze chitosan to primarily D-GlcN and chitobiose (Guo et al. 2019). To improve industrial chitosanase application, researchers have used carbohydrate-binding module fusion technology to efficiently immobilize GH46 chitosanase (Lin et al. 2019). The stability of the immobilized enzyme is superior to that of the natural enzyme, and three chitosan products with different molecular weights can be produced via the optimized reaction (Lin et al. 2019). However, chitosan has a general limitation as a substrate because it is not completely deacetylated, which results in a nonuniform degree of acetylation of the chitosanase hydrolysate. The single DP and the degree of acetylation of the product components have a strong effect on the identification of the activity of the bioactive components of COS. Two partially acetylated chitotrioses (*N*-acetylchitotriose and *N*,*N*′-diacetylchitotriose) were produced to study the relationship between activity and acetylation. The antioxidant activities of two partially acetylated shell trisaccharides and virgin trisaccharides were further studied. *N*,*N*′-diacetylchitotriose, with a high degree of acetylation, has the highest antioxidant activity (Li et al. 2013). Furthermore, COS, with a 50% degree of acetylation, was the most effective at alleviating salt stress in wheat seedlings (Zou et al. 2015). These results indicated that the activity of COS was closely related to its degrees of acetylation and polymerization.

However, despite the presence of the chitinase gene in bacterial and fungal genomes, the ability to hydrolyze insoluble chitin has been identified in only a few species, such as *Serratia mucilis* and *Bacillus*, *Pseudomonas* and *Streptomyces* species (Hara et al. 2013; Sorokin et al. 2014; Durairaj et al. 2017; Ilangumaran et al. 2017; Moon et al. 2017). Chitinase is a specific hydrolase that directly hydrolyzes chitin to produce fully acetylated COS. *Salinivibrio* sp. BAO-1801 was isolated from the fermentation broth of salted shrimp, and its chitinase was characterized (Le and Yang 2018). The main product of this enzyme is acetylchitobiose. The binding of different domains to insoluble chitin was studied by NMR spectroscopy (Takashima et al. 2018). The CBM18 domain hydrolyzed insoluble chitooligosaccharide better than the GH19 domain.

Although the chitinases produced by microorganisms have made significant contributions to the transformation of shrimp and crab shell wastes, their molecular and ecological roles in industrial applications have not been fully explored (Nazari et al. 2011). To study the degradation mechanism of enzymes with specific effects produced by microorganisms, whole-genome sequencing technology is widely used. *Bifidobacterium choerinum* FMB-1, which can degrade resistant starch, was subjected to whole-genome analysis, and 11 protein-coding genes related to α-glucan degradation were found (Jung et al. 2018). Cellulase-producing *Paenibacillus lautus* BHU3 was subjected to whole-genome analysis, and 143 glycoside hydrolase (GH) genes were discovered. These genes may play a vital role in enhancing cellulolytic attributes (Yadav and Dubey 2018).

Currently, few draft genome sequencing data sets are available for chitinase-producing strains capable of degrading insoluble chitin (Sorokin et al. 2014). Therefore, improving the pool of information via whole-genome sequencing analyses of different strains that produce chitinase will aid in elucidating the genomic basis of chitin decomposition activity and the utilization of degraded chitin. In our previous study, we selected a strain from fermented shrimp paste that could decompose chitin to COS and identified it as *Streptomyces diastaticus* CS1801 (Xu et al. 2019). In this study, whole-genome sequencing of the chitinase-producing *S. diastaticus* strain CS1801 was performed to describe specific genomic information regarding chitin degradation activity. The possibility of directly degrading shrimp and crab shells to produce COS was studied.

## Materials and methods

### Determination of the ability of CS1801 to transform COS

The bacteria were isolated from shrimp paste and stored in the China Center for Type Culture Collection (CCTCC, Wuhan, China). The isolate was classified as *Streptomyces diastaticus* CS1801, and the accession number is CCTCC No. M2018263. Using chitin colloid, GlcNAc or Chinese *Eriocheir sinensis* shell powder as the sole carbon source, the production of five kinds of COSs (DP = 1–5) was evaluated in the fermentation broth of CS1801 with ultra-high-performance liquid chromatography-mass spectrometry (UPLC-MS, Waters, Massachusetts, United States) after 5 days of fermentation. To prepare the colloidal chitin, flaked chitin was shredded, and 10 g of chitin powder was slowly added to 200 mL of concentrated hydrochloric acid and stirred quickly. After the colloidal chitin was dissolved completely, the impurities were removed by glass cotton filtration, and the solution was added to 1000 mL of distilled water. A precipitate was obtained by centrifugation and washed with distilled water for neutralization. The fermentation medium was prepared as follows: solution A consisted of 1.4 g/L K_2_HPO_4_, 0.6 g/L KH_2_PO_4_, 1 g/L MgSO_4_∙7H_2_O, 10 g/L NaCl, and 20 g/L (NH_4_)_2_SO_4_ in 1000 mL of deionized water at pH 6.5, and solution B consisted of 10 g/L sole carbon source at pH 6.5. The two solutions were mixed in equal volumes before use.

The standards were 0.1429 mg/mL GlcN, 0.1429 mg/mL shell disaccharide ((GlcN)_2_), 0.1429 mg/mL shell trisaccharide ((GlcN)_3_), 0.1429 mg/mL shell tetrasaccharide ((GlcN)_4_) and 0.1429 mg/mL shell pentasaccharide ((GlcN)_5_). The COS standard product was purchased from Shanghai Huich Biotech Inc. The experimental conditions for UPLC-MS followed those published in an article by our laboratory in 2019 (Xu et al. 2019). The conversion rate was the ratio of the total COS concentration (DP = 1–5) to the sole carbon source concentration.

$$ {\text{Conversion rate}} = \frac{{COS({\text{DP}} = 1 - 5)({\text{mg}}/{\text{L}})}}{{5000({\text{mg}}/{\text{L}})}} \times 100{\text{\% }} $$

### Whole-genome sequencing assembly

Genomic DNA of *S. diastaticus* CS1801 was extracted using a Bacterial Genomic DNA Extraction Reagent Kit (Sangon Biotech, Bioengineering Biotechnology (Shanghai) Co., Ltd., Shanghai, China).

Sequencing was performed using an Illumina HiSeq™ second-generation sequencer (Illumina, Inc., Delaware, United States), and the linker sequences and low-mass bases in the reads were removed using Trimmomatic. Nonamplified long DNA fragments were sequenced using a PacBioRS II third-generation sequencer (PacBio, Pacific Biosciences of California, Inc., Delaware, United States). The linker sequences and low-mass bases in the reads were removed after sequencing and according to the estimated genome size. The data were pooled and analyzed until an estimated 40X coverage of the genome was obtained. Canu was used to splice three generations of single-molecule sequencing data, followed by second-generation sequencing data. The scaffold complement GAP was obtained by splicing with GapFiller, and finally, the sequence data were corrected by PrInSeS-G to modify editing errors and the insertion of small fragments during the splicing process.

### Homologous gene alignment

The common genes and unique genes of CS1801 and its near-source strains were obtained by the pangenome analysis pipeline (PGAP) for further analysis. The genomic information and gene sequences of the near-source strains are shown in Table 1. First, PGAP was used to collect the protein sequences of all strains for BLAST alignment. According to the BLAST alignment results, the similarity between different proteins was determined, and similar genes were assigned to same-ortholog clusters. A homologous gene present in all samples was used as a core gene. Then, the shared gene was removed, a nonconsensus gene was obtained, and the specific gene was a gene uniquely possessed only by the tested sample. All nonconsensus genes were combined with a consensus gene as a pangene. A phylogenetic tree was constructed based on the neighbor-joining clustering results from homologous genes and pangenome analysis.

**Table 1:** List of strains closely related to *Streptomyces diastaticus* CS1801

Tax ID	Name	Assembly ID	Level	Distance
1638939	*Streptomyces* sp. KE1	ASM101459v1	Contig	5.9e−03
463642	*Streptomyces* sp. F-1	*Streptomyces*_sp_F1_v3	Scaffold	7.9e−03
579932	*Streptomyces* sp. FXJ7.023	*Streptomyces* sp. FXJ7.023	Contig	8.8e−03
682181	*Streptomyces* sp. SA3_actF	ASM17921v1	Contig	9.8e−03
1463876	*Streptomyces* sp. NRRL F-6628	ASM72145v1	Contig	0.01
1428626	*Streptomyces malaysiense*	ASM98088v2	Contig	0.01
996637	*Streptomyces griseoaurantiacus* M045	ASM20460v1	Contig	0.01
1286094	*Streptomyces aurantiacus* JA 4570	STRAU	Contig	0.01
889487	*Streptomyces* sp. S4	ASM29771v1	Scaffold	0.01
42239	*Streptomyces sampsonii*	ASM170419v1	Complete genome	0.01
1463837	*Streptomyces* sp. NRRL B-3253	Doro.v1.0	Scaffold	0.01
545123	*Streptomyces silaceus*	ASM141974v1	Scaffold	0.01
412968	*Streptomyces* sp. M10	ASM80053v1	Scaffold	0.01
457425	*Streptomyces albus* J1074	ASM35952v1	Complete genome	0.01
1571532	*Streptomyces* sp. CNQ431	GCF_000797385.1	Complete genome	0.01

### Genome prediction and annotation

Rapid prokaryotic genome annotation (Prokka) was used to predict the assembly results of the gene components, and the obtained genes were submitted to Clusters of Orthologous Groups (COG) of proteins (Tatusov et al. 2000), Gene Ontology (GO), and Kyoto Encyclopedia of Genes and Genomes (KEGG) databases (Kanehisa and Goto 2000) and compared to obtain functional annotation information.

### CAZy carbohydrase analysis

The protein sequences inferred from the whole-genome sequence were aligned with the carbohydrate active enzyme (CAZy) database (http://www.cazy.org/) using HMMER3 to obtain the corresponding carbohydrate-active enzyme annotation information (Lombard et al. 2014). The screening condition was E-value < 1e^−5^.

### Drug resistance functional annotation

The protein sequences inferred from the whole-genome sequence were compared with the Comprehensive Antibiotic Resistance Database (CARD) by BLAST (McArthur et al. 2013), and the annotation information for each gene and its corresponding drug resistance function was combined to obtain the annotation result.

### NCBI registration number

The whole-genome sequence data of *S*. *diastaticus* CS1801 (6.9 Mb) were deposited in the NCBI database with the accession number SUB5461644.

## Results

### Analysis of COS conversion

The fermentation broth with colloidal chitin as the sole carbon source contained 6.865 mg/L (GlcN)_2_, 10.949 mg/L (GlcN)_3_, 15.603 mg/L (GlcN)_4_, and 20.833 mg/L (GlcN)_5_, and its total conversion rate was 1.085%. The fermentation broth with GlcNAc as the sole carbon source contained 299.753 mg/L GlcN, 5.010 mg/L (GlcN)_3_, 9.894 mg/L (GlcN)_4_, and 23.398 mg/L (GlcN)_5_, and its total conversion rate was 6.761%. The fermentation broth with crab shell powder as the sole carbon source contained 1.670 mg/L (GlcN)_3_, 5.988 mg/L (GlcN)_4_, and 34.262 mg/L (GlcN)_5_, and its total conversion rate was 0.838%. These results are summarized in Table 2.

**Table 2:** Results for transformation of COS by *Streptomyces diastaticus* CS1801

Carbon source	Total conversion rate (%)	COS (mg/L)				
		GlcN	(GlcN)_2_	(GlcN)_3_	(GlcN)_4_	(GlcN)_5_
Colloidal chitin	1.085	0	6.865	10.949	15.603	20.833
GlcNAc	6.761	299.753	0	5.010	9.894	23.398
Crab shell	0.838	0	0	1.670	5.988	34.262

### Complete genome of *Streptomyces diastaticus* CS1801

**Fig. 1:**
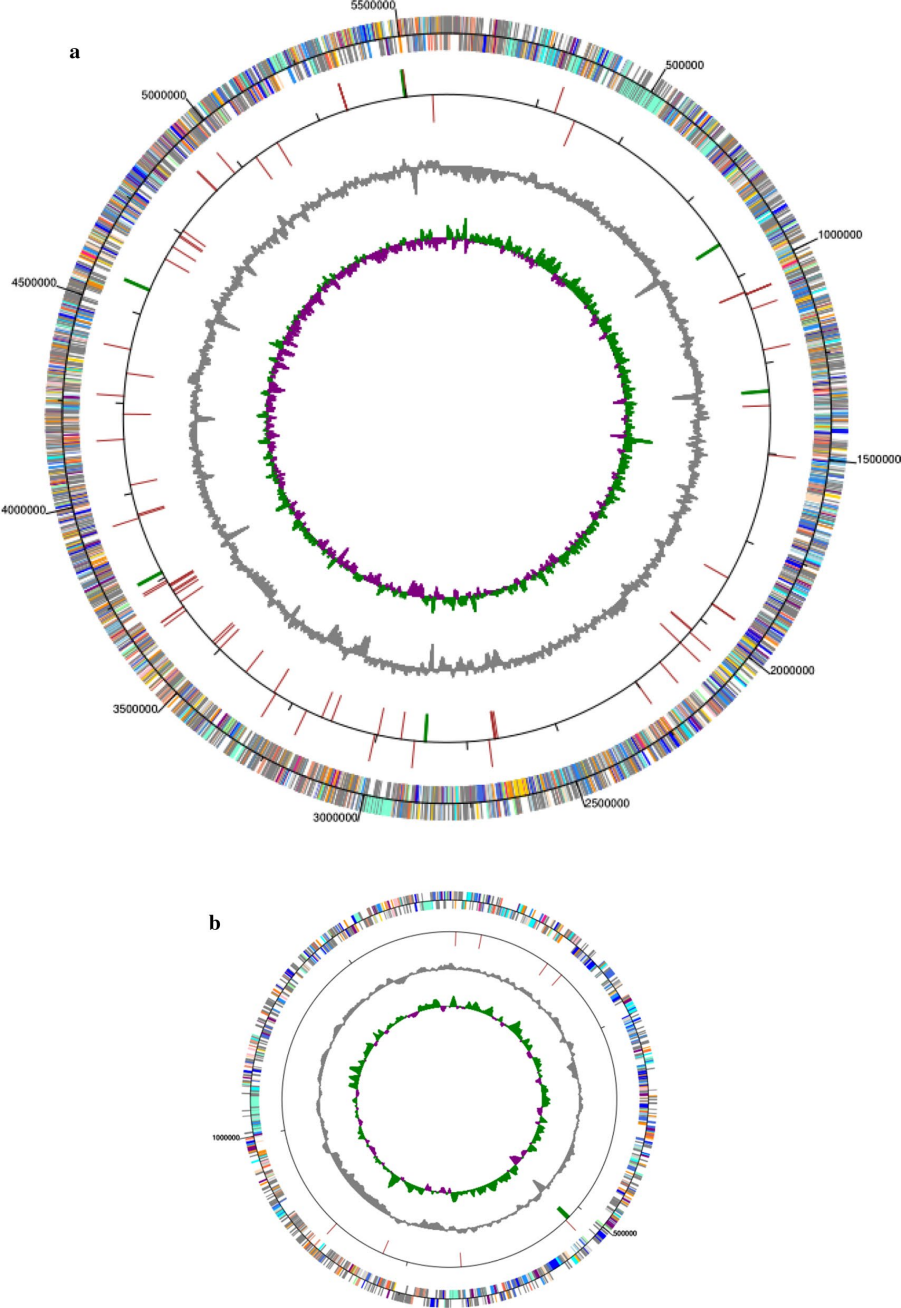
Circular map of the *Streptomyces diastaticus* CS1801 genome. **a** Chromosome; **b** plasmid. From the outside to the inside: the first circle represents the COG database-annotated genes, with the color corresponding to the COG-annotated letter; the second circle represents RNA; the third circle is the GC content (the peak level reflects the GC amount), with the outer circle representing the sense chain and the inner circle representing the antisense chain; and the innermost circle reflects the asymmetry of the GC content

**Fig. 2:**
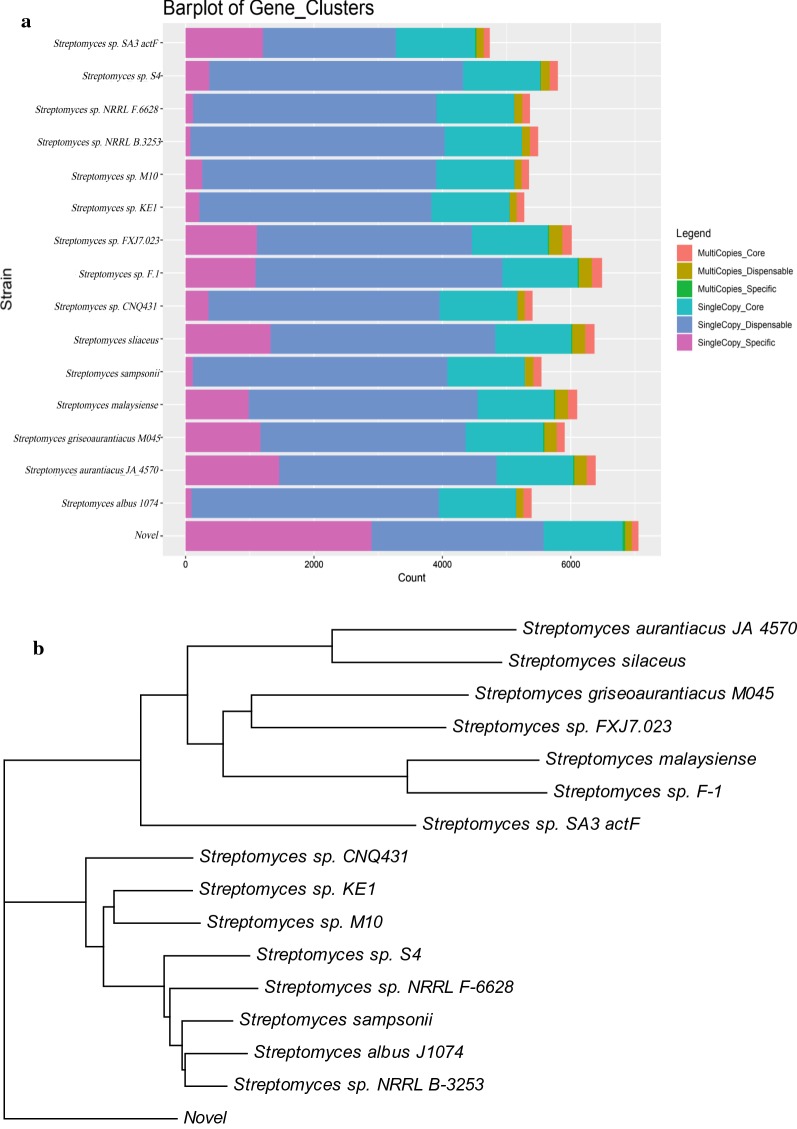
**a** Histogram of the number of homologous genes. **b** Phylogenetic tree based on pangenes constructed by neighbor-joining clustering

**Fig. 3:**
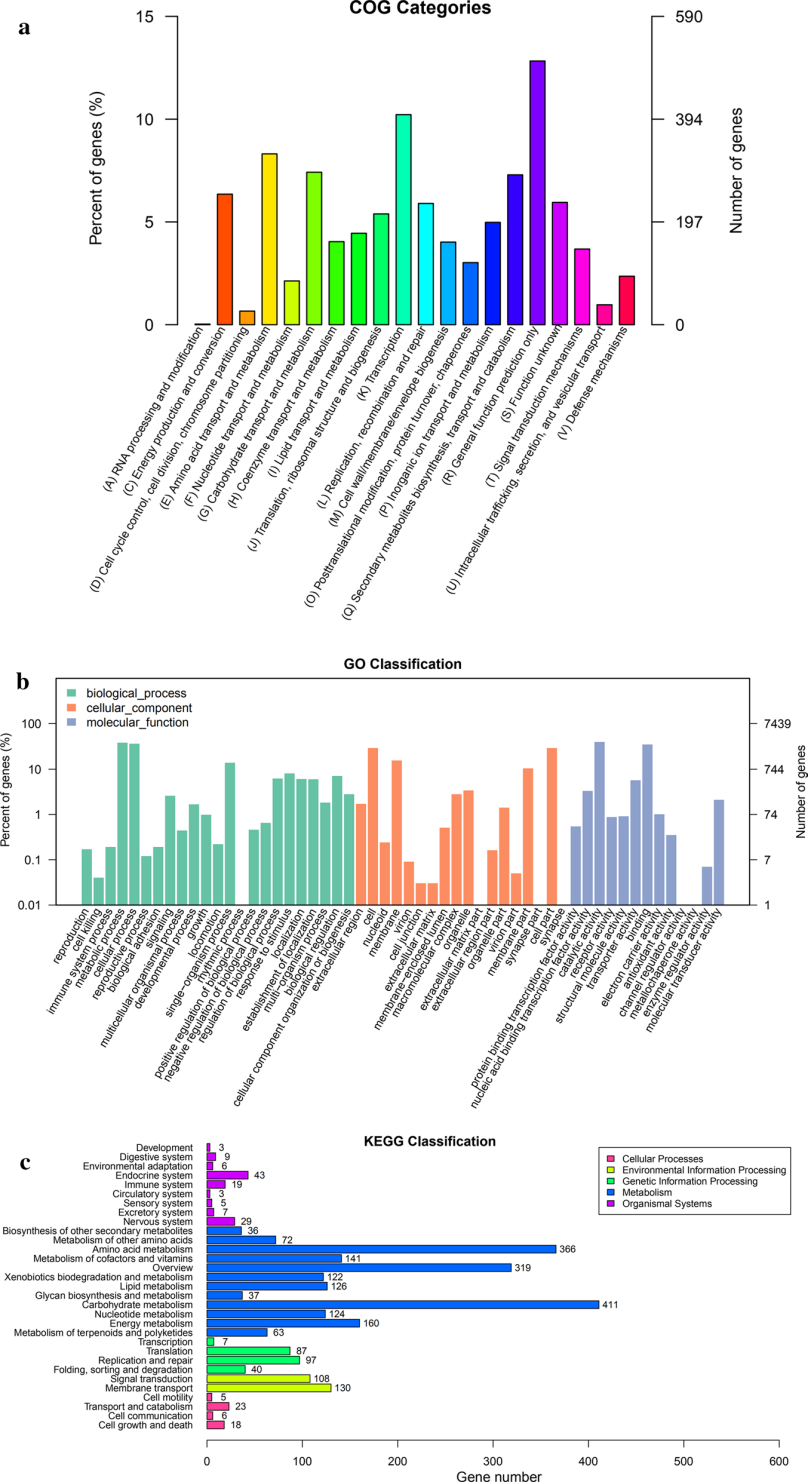
**a** COG annotation distribution histogram; **b** GO annotation distribution histogram; **c** KEGG pathway classification histogram

Genome-wide analysis was performed to decipher the full set of genes involved in chitin degradation. The complete genome of *S. diastaticus* CS1801 is composed of two separated circular sequences: one is a 5,611,479-bp chromosome with a 73% GC content, and the other is a 1,388,284-bp plasmid with a 73% GC content (Fig. 1). The genome consists of 7584 protein-coding genes and 90 tRNA and 21 rRNA operons. The whole-genome sequence of *Streptomyces diastaticus* CS1801 was compared with that of a near-source strain, and the number of homologous genes was counted (Fig. 2a). Based on a comparative analysis of the ubiquitous gene set consisting of genes and nonconsensus genes, the genome has 2898 unique single genes, which is a much higher number than that of closely related strains. Phylogenetic tree analysis showed that *S. diastaticus* CS1801 is orthologous to related strains and that the strains are derived from a common ancestor (Fig. 2b). A total of 2633 unigenes were annotated in the COG database and were assigned to 14 functional groups (Fig. 3a). Among the groups, transcription (10.22%), amino acid transport and metabolism (8.31%), and carbohydrate transport and metabolism (7.42%) were the most abundant, while the least enriched functional group was RNA processing and modification (0.03%). The genes in the carbohydrate transport and metabolism functional group are closely related to the degradation of chitin to COS. A total of 9865 unigenes were classified into the biological process category, 6963 unigenes were classified into the cellular component category, and 6656 unigenes were classified into the molecular function category (Fig. 3b). The largest functional groups in the biological process category were metabolic process and cellular process. In the cellular component category, the largest functional groups were cell and cell part, and the largest functional groups in the molecular function category were catalytic activity and binding. A total of 2667 unigenes were annotated in the KEGG database (Fig. 3c), of which 411 unigenes were classified as carbohydrate metabolism genes, 37 unigenes were classified as glycan biosynthesis genes, and 126 unigenes were classified as lipid metabolism genes.

### CAZy carbohydrase analysis

The results (protein count) showed that CS1801 has 90 GHs, 54 glycosyl transferases (GTs), 53 carbohydrate esterases (CEs), 29 auxiliary activities (AAs), 22 carbohydrate-binding modules (CBMs) and 6 polysaccharide lyases (PLs). Extended CAZyme analysis indicated that the GH family was divided into 103 GH subfamilies (Table 3). Among the GHs, the major subcategory was GH13, followed by GH15, GH18 and GH23. Chitinases are present mainly in GH18 and GH19 (Suma and Podile 2013). *N*-Acetylglucosaminidase can split the β-(1,4)-glycosidic bond of *N*,*N*′-diacetylchitobiose ((GlcNAc)_2_) to generate GlcNAc (Haran 1996). *N*-Acetylglucosaminidase plays an important role in the degradation of chitin and is broadly distributed in GH3, GH20, GH83 and GH116 (Ferrara et al. 2014). This scenario is also true for CS1801, which harbors 7 genes from GH18 and 2 genes from GH19, including chitinases. Among GTs, the major subcategory was GT87, followed by GT43. Among CBMs, the major category was CBM13, followed by CBM5, CBM12 and CBM32. Among CEs, the major subcategory was CE7, followed by CE10 and CE4. Chitin deacetylase converts chitin to chitosan and is a member of the carbohydrate esterase family 4 (CE4) defined in the CAZy database (Park et al. 2010). In the CS1801 genome, CBM2, CBM5, CBM12, CBM37 and CBM50, which have affinity for chitin, were also detected (http://www.cazy.org/Carbohydrate-Binding-Modules.html: Park et al. 2010).

**Table 3:** Carbohydrate-active enzyme counts of *Streptomyces diastaticus* CS1801

CAZy	Count	CAZy	Count	CAZy	Count	CAZy	Count
AA1	2	CE1	15	GH39	1	GT7	1
AA3	12	CE3	6	GH42	1	GT9	2
AA4	5	CE4	10	GH46	1	GT12	3
AA5	3	CE7	12	GH55	1	GT2_	2
AA6	1	CE8	1	GH57	2	GT20	1
AA7	7	CE9	9	GH63	2	GT21	5
AA8	7	CE10	11	GH64	1	GT27	5
AA10	2	CE14	4	GH65	4	GT28	4
CBM2	2	GH1	5	GH74	1	GT35	2
CBM3	1	GH2	3	GH77	1	GT39	1
CBM5	3	GH3	3	GH78	3	GT41	11
CBM6	1	GH4	1	GH81	2	GT45	2
CBM12	3	GH5	2	GH84	4	GT50	1
CBM13	4	GH6	3	GH87	1	GT51	4
CBM16	1	GH13	11	GH93	1	GT76	1
CBM20	1	GH15	7	GH95	2	GT81	5
CBM32	3	GH16	1	GH103	2	GT83	3
CBM36	1	GH18	7	GH109	4	GT87	14
CBM37	1	GH19	2	GH114	1	PL6	1
CBM42	1	GH20	3	GH119	4	PL9	1
CBM47	1	GH23	7	GH135	1	PL10	1
CBM48	2	GH25	4	GT1	4	PL11	2
CBM50	2	GH31	1	GT2	14	PL22	1
CBM51	1	GH33	2	GT4	11		
CBM66	1	GH35	1	GT5	11		

### CARD analysis

A total of 63 AR proteins in *S. diastaticus* CS1801 were predicted, including DNA gyrase subunit B, the daunorubicin doxorubicin resistance ATP-binding protein DrrA, a β-lactamase precursor, virginiamycin B lyase, the quaternary ammonium compound-resistance protein SugE and other antibiotic resistance proteins. In addition, CS1801 has two kinds of resistance proteins, aminoglycoside/hydroxyurea antibiotic resistance kinase (EC 2.7.1.72) and UDP-GlcNAc 1-carboxyvinyltransferase (EC 2.5.1.7), which may be closely related to COS inhibition in the late fermentation stage.

### Analysis of enzymes involved in the metabolism of chitin

Both the fermentation products and the whole-genome data of CS1801 aid in elucidating the chitin degradation, metabolism and synthesis mechanisms. The most important phenotypic property of the CS1801 strain is its ability to efficiently hydrolyze chitin and utilize it as a growth substrate. CS1801 contains various genes encoding specific enzymes with chitin hydrolysis activity, such as 9 chitinases, 5 *N*-acetylglucosaminidases, 10 chitin deacetylases, 4 β-galactosidases, 7 β-glucosidases and 1 chitosanase. In addition, CS1801 contains a variety of enzymes that are thought to be involved in chitin synthesis. Details of the enzymes are listed in Table 4.

**Table 4:** Genes encoding enzymes involved in chitin metabolism

Number	Predicted genes	EC	Activity
1	PROKKA01070, 02558, 05523, 05524, 05525, 05527, 05918, 07311, 07336	EC: 3.2.1.14	Chitinase
2	PROKKA02420, 05378, 02684, 05073, 05074	EC: 3.2.1.52	*N*-Acetylglucosaminidase
3	PROKKA00813, 01449, 02821, 03087, 05050, 05463, 06475, 06684, 06832, 07353	EC: 3.1.1.72/EC: 3.5.1.41	Acetyl xylan esterase/chitin deacetylase
4	PROKKA05224, 05225, 06460, 07087	EC: 3.2.1.23	β-Galactosidase
5	PROKKA00858, 02420, 02486, 04829, 04830, 05378, 06458	EC: 3.2.1.21	β-Glucosidase
6	PROKKA01722	EC: 3.2.1.132	Chitosanase
7	PROKKA01398, 02174	EC: 2.7.1.12	Glucokinase
8	PROKKA02470, 02853, 04321	EC: 2.6.1.16	GlcN-fructose-6-phosphate aminotransferase (isomerization)
9	PROKKA02857	EC: 5.4.2.10	Phosphoglucosamine mutase
10	PROKKA04318	EC: 3.5.99.6	GlcN-6-phosphate deaminase
11	PROKKA04700	EC: 2.3.1.157/EC: 2.7.7.23	GlcN-1-phosphate *N*-acetyltransferase/UDP-N-GlcNAc pyrophosphorylase
12	PROKKA00223, 01062, 01446, 04349, 04925, 04958, 05020, 05024, 05028, 05050, 05156, 05590, 06114, 06196	EC: 2.4.1.12/EC: 2.4.1.16	Cellulose synthase/chitin synthase

## Discussion

**Fig. 4:**
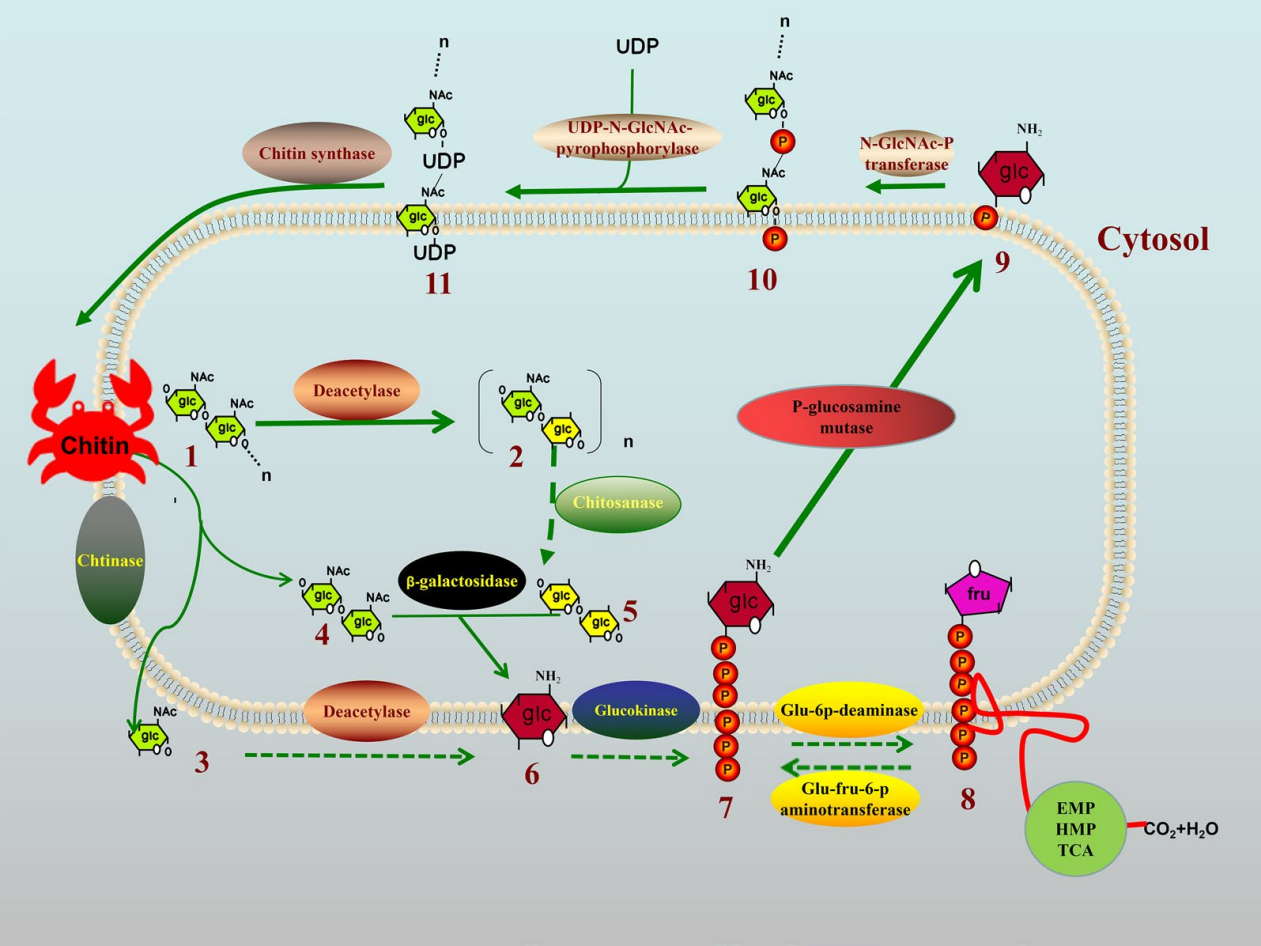
Proposed chitin degradation and synthesis pathways of CS1801. 1: Chitin; 2: chitosan; 3: GlcNAc; 4: (GlcNAc)_2_; 5: (GlcN)_2_; 6: GlcN; 7: 6-phosphate-GlcN; 8: 6-phosphate-fructose; 9: GlcN-1-phosphate; 10: *N*-GlcNAc-1-phosphate; 11: UDP-N-GlcNAc

The results of fermentation revealed that *S. diastaticus* CS1801 can convert the chitin derived from crab shells, colloidal chitin and *N*-acetylglucosamine to COS. To explore the mechanism of the chitin degradation process, we performed a genome-wide analysis of the CS1801 strain and found a large number of genes related to enzymatic hydrolysis of COS, the most important of which is chitinase. The predicted chitin degradation and synthesis pathways of CS1801 are proposed in Fig. 4. CS1801 degrades chitin through three predicted pathways as follows: (i) endochitinase hydrolyzes the insoluble form of chitin to water-soluble oligomers, especially (GlcNAc)_2_, and then, (GlcN)_2_ is generated from (GlcNAc)_2_ by chitin deacetylase. (ii) GlcNAc is ultimately produced from chitin by exochitinase, and GlcNAc is transformed to GlcN by chitin deacetylase. (iii) Chitosan is generated from chitin by chitin deacetylase and then is transformed to COS by chitosanase (Seki et al. 2019). All of the (GlcN)_2_ molecules are transformed to GlcN by β-galactosidase or β-glucosidase. β-Galactosidase and β-glucosidase split the β-(1,4)-glycosidic bond of (GlcN)_2_ to generate GlcN (Chinchetru et al. 1989; Sorokin et al. 2014). Then, GlcN is transformed to 6-phosphate-GlcN by glucokinase (Sorokin et al. 2014), GlcN-6-phosphate deaminase (EC 3.5.99.6) transforms 6-phosphate-GlcN to 6-phosphate-fructose, and finally, CO_2_ and H_2_O are formed by the action of the EMP, HMS and TCA cycles. In this unique manner, CS1801 can synthesize chitin. First, GlcN-6-phosphate-fructose aminotransferase (isomerization) transforms 6-phosphate-fructose to 6-phosphate-GlcN (Leriche et al. 1997). Then, 6-phosphate-GlcN is transformed to GlcN-1-phosphate by phosphoglucosamine mutase (Shimazu et al. 2012). *N*-GlcNAc-1-phosphate is formed by GlcN-1-phosphate *N*-acetyltransferase, which transforms GlcN-1-phosphate to *N*-GlcNAc-1-phosphate (Zhang et al. 2015). Next, UDP-N-GlcNAc is formed by UDP-N-GlcNAc pyrophosphorylase, which can transform *N*-GlcNAc-1-phosphate to UDP-N-GlcNAc (Ullrich and van Putten 1995). Finally, chitin is produced by poly-β-1,6-*N*-acetyl-d-GlcN synthase (chitin synthase) (Sburlati and Cabib 1986).

CS1801 can hydrolyze colloidal chitin to (GlcN)_2_, (GlcN)_3_, (GlcN)_4_ and (GlcN)_5_. It can also synthesize (GlcN)_3_, (GlcN)_4_ and (GlcN)_5_ from GlcNAc. According to these findings, CS1801 has the complete metabolic pathway for chitin, including hydrolysis and synthesis. *Streptomyces coelicolor* A3 (2) is a well-studied *Streptomyces* strain that has a known whole-genome sequence (Bentley et al. 2002) containing 13 chitinase genes (some of which are putative). However, CS1801 has 9 chitinases, 1 chitosanase and 7 potential chitinases (PROKKA00833, 02567, 02568, 05526, 05528, 06351, and 07303) with binding domains associated with chitin degradation. *Chitinivibrio alkaliphilus* gen. nov., sp. nov. is a novel extremely haloalkaliphilic anaerobe that has fewer than 5 chitinases (Sorokin et al. 2014). CS1801 has more chitinases than other strains and a more complete chitin metabolism pathway.

CS1801 can directly convert untreated crab shell waste to COS with a molecular weight less than 1000 Da, which has good commercial application value (Chen et al. 2003, 2005). To further study the mechanism of CS1801-mediated chitin degradation, the enzymes in the reaction pathway will be cloned and expressed in subsequent work, and directed evolution can be used to control the degree of chitin hydrolysis. Finally, a COS with single polymerization and acetylation can be obtained with improved purity. In general, CS1801 is a very interesting strain, and CS1801 or its enzymes have the potential to produce COSs. This study will lay a good foundation for industrial COS production and increase the added value of waste, such as crab shells.

## Data Availability

The data used to support the findings of this study are included within the article.

## Abbreviations

COS: Chitooligosaccharide
COG: Cluster of Orthologous Groups database
GO: Gene Ontology functional database
KEGG: Kyoto Encyclopedia of Genes and Genomes pathway database
CAZy: Carbohydrate-active enzymes
CARD: Comprehensive Antibiotic Resistance Database
AA: Auxiliary activity
GH: Glycosyl hydrolase
CBM: Carbohydrate-binding module
CE: Carbohydrate esterase
GT: Glycosyl transferase
PL: Polysaccharide lyase
GlcNAc: *N*-acetylglucosamine
GlcN: Glucosamine
